# Development of an Evidence-Informed and Codesigned Model of Support for Children of Parents With a Mental Illness— “It Takes a Village” Approach

**DOI:** 10.3389/fpsyt.2021.806884

**Published:** 2022-01-31

**Authors:** Melinda Goodyear, Ingrid Zechmeister-Koss, Annette Bauer, Hanna Christiansen, Martina Glatz-Grugger, Jean Lillian Paul

**Affiliations:** ^1^School of Rural Health, Monash University, Melbourne, VIC, Australia; ^2^Emerging Minds, National Workforce Centre for Child Mental Health, Hilton, SA, Australia; ^3^Austrian Institute of Health Technology Assessment, Vienna, Austria; ^4^Department of Health Policy, Care Policy and Evaluation Centre, London School of Economics and Political Science, London, United Kingdom; ^5^Department of Psychology, Clinical Child and Adolescent Psychology, Philipps University Marburg, Marburg, Germany; ^6^Mental Health Research Program, The Village, Ludwig Boltzmann Gesellschaft, Innsbruck, Austria; ^7^Division of Psychiatry I, Department of Psychiatry, Psychotherapy and Psychosomatics, Medical University Innsbruck, Innsbruck, Austria

**Keywords:** codesign, family focused, strength-based, children, parents with mental illness, family intervention, prevention, early intervention

## Abstract

Providing support to parents and their children to help address the cycle of intergenerational impacts of mental illness and reduce the negative consequences for children is a key focus of selective prevention approaches in public mental health. However, a key issue for children of parents with a mental illness is the lack of access to early intervention and prevention support when needed. They are not easily identifiable (until presenting with significant mental health issues of their own) and not easily accessing the necessary support that address the complex interplay of parental mental illness within families. There are significant barriers to the early identification of these children, particularly for mental health care. Furthermore, there is a lack of collaborative care that might enhance identification as well as offer services and support for these families. The “It takes a Village” project seeks to improve mental health outcomes for children through the co-development, implementation and evaluation of an approach to collaborative practice concerned with the identification of families where a parent has a mental illness, and establishing a service model to promote child-focused support networks in Austria. Here we describe the development of service delivery approach for the “It takes a Village” project that aims to improve identification and support of these children within enhancements of the existing service systems and informal supports. The paper describes the use of codesign and other implementation strategies, applied to a research setting, with the aim of impacting the sustainability of workforce reform to achieve lasting social impact. Results highlight the steps involved in translating evidence-based components, local practice wisdom and lived experience into the “It takes a Village” practice model for Tyrol, Austria. We highlight through this paper how regional context-specific solutions are essential in the redesign of care models that meet the complex needs of children of parents with a mental illness. Service system and policy formation with local and experienced stakeholders are also vital to ensure the solutions are implementation-ready, particularly when introducing new practice models that rely on organizational change and new ways of practice with vulnerable families. This also creates a solid foundation for the evaluation of the “It take a Village” approach for children of parents with a mental illness in Austria.

## Introduction

International studies estimate that one in four children currently grows up with a parent with mental illness worldwide (1). Children whose parents have mental illness have an increased risk of developing behavioral, academic, and/or mental health problems due to a range of genetic, environmental, and psychosocial factors (2). A key issue for these children is that they are often considered “invisible” from view of the existing service system in accessing early intervention support (3). Increased engagement with these children from services that may come into contact with their families can help provide supports to promote the healthy development of these children (4).

Mental illness typically occurs within families, impacting parents, children, and the whole family situation (5). Population estimates indicate that over 50% of people with a lifetime diagnosis of mental illness are parents (6), and worldwide, between 12 and 45% of adults attending adult mental health services are parents (7). These parents face similar parenting issues as all parents and while not all parents with a mental illness struggle, there are many that do, often due to issues such as poverty and social isolation usually associated with mental illness (8). Furthermore, because of the increased likelihood of stigmatization and discrimination accompanying a mental illness, these families may face greater challenges accessing support. This, in combination with a lack of visibility for early intervention support, may explain why less than one in six children are currently receiving support for emerging mental health issues at any one time (9, 10).

Family-focused service delivery in mental health services is a model that views the person with the mental illness in the context of their family relationships (e.g., being a parent) (4, 11). Family focused practice, targeting support toward supporting parenting and child well-being, has been a promising selective prevention strategy as a way to enhance public mental health at the population level (12). This type of approach focusses on supporting families to buffer against the impacts of mental illness on all family members, including children (13, 14). However, this type of service delivery is not as common in services who might be coming into contact with these parents and their families (15). In adult mental health, for example, a change to this type of service delivery is slow, as it is in conflict with the predominant medicalised individual client care model, and enhanced by limiting supportive administrative structures to encourage family focused practice (16–19).

Providing targeted intervention support to parents and their children can help break the cycle of intergenerational transmission of mental illness and improve outcomes for children of parents with a mental illness (20). Several approaches to address the intergenerational impacts have been outlined worldwide (21). Early intervention programs targeting parents with a mental illness have been shown to be effective in reducing vulnerability of young people to mental illness or negative social outcomes (22). A meta-analysis has shown that intervention with families can reduce the incidence of children developing similar mental health issues by up to 40% (22). Analysis of randomized control trials found that individual, group and family-based interventions were effective in reducing internalizing behavior and, to a lesser extent, externalizing behavior in children of parents with a mental illness (22, 23).

Interventions targeting parental behavior or parent-child interactions have typically shown small but significant positive outcomes on sensitivity and responsiveness between parents and children (24). Parenting support models have been developed as an early intervention approach addressing parenting behavior and understanding of child development through social learning models (25). Adaptation of parent support programs is commonplace though, to respond to the fear of negative judgement and stigma and shame that can accompany mental illness and/or co-occurring substance misuse for parents (26).

As mentioned, there are challenges for children experiencing a vulnerability to their mental health in accessing early intervention support (2, 4). They are not easily identifiable (until in significant need of their own treatment) and do not easily access the necessary support to address the impact of mental illness within families (3). Furthermore, support in adult focused services has typically been focused on engaging the parent in the care of the child, with limited consultation regarding the nature of that support that addresses the needs and listens to the “voice” of the child (27).

A need still exists for systemic change which emphasizes the early identification and prevention of risk factors for children's mental health (28–31). Making these children visible involves both direct support to children and parents focussing on improving behavioral outcomes; as well as a need to draw on strategies to promote motivation to change for those families deeply affected by systemic disempowerment (4, 5, 32, 33). Whole of family approaches and integrated models need to be considered moving forward to address the multiple and complex needs of these families, and addresses the influences that affect generations with mental health challenges (13, 34–37).

There is emerging evidence for the role of collective impact and integrated models supporting parents with mental health challenges and their children. In Finland, a brief intervention model such as Let's Talk About Children (38), helping parents with mental illness to support the everyday life of the child, has shown effectiveness when implemented across adult, child, and family focused services in a region. The program has been shown to improve outcomes for children and parents in terms of emotional symptoms, parental self-efficacy, and result in a significant decline in child protection referrals when the intervention is implemented across all service systems interacting with families (39).

We have recently developed a similar integrated early intervention model using codesign and open innovation in science methods in Austria (40). Our international, multi-disciplinary-led initiative takes the concept of “raising the village to raise the child” and applied it to an early identification and collaborative support approach to improve outcomes for children of parents with a mental illness and their families (“It takes a Village” practice approach) (3). The approach is aimed at improving early identification of children and adolescents whose parents have a mental illness (sensitive identification; SENSE) and enhancing the support networks around the child and their family by increasing their informal and formal resources (Collaborative Village Approach; CVA). The project aims to focus on the children's perspective (of their support network) and design an approach that is collaborative, strength-based, and offers support to the family in the region of Tyrol, Austria (8).

This paper describes this early intervention model in detail. In doing so, we describe the process of development of the practice approaches—SENSE and CVA through an extensive scoping and codesign process to develop evidence informed practice approaches that not only draw on practice wisdom and local context knowledge, but also draw on the international research on interventions for these children and their families. The co-design approach utilized in the Village project is influenced by the notion of participatory research, whereby researchers work together with key stakeholders with a good understanding of the local system, to use their collective experiences and creativity to co-create a new product, practice or new way of addressing a local issue (41, 42). This approach benefits from the value it places on sharing the production of knowledge across disciplines or across contexts, as a way to enhance the usability and social relevance of the knowledge generated, particularly for community-based or health-based services (41, 43).

Based on the premise of participatory design, the development of knowledge in this way in partnership with those who will use it, is believed to facilitate knowledge translation and support the integration of the practice approaches in the real world setting for evaluation. The translation of evidence into the routine delivery of family focused practice supporting families where a parent has a mental illness continues to be a significant challenge in this field (44). Here we invited community stakeholders including people living with a mental illness or their children and professionals, to participate in a creative group process with the goal of designing new practice approaches for adult mental health and other support services to provide support for children of parent with mental illness (3). The rationale behind the idea of designing practice approaches in a participatory manner is that the approaches better suit the context, are accepted and valued by stakeholders and are more sustainable than producer-push approaches (45).

## Materials and Methods

This paper continues on from the protocol paper published in Volume 1 in this special interest topic (3) by showcasing the results of the codesign process, in which the development of the practice approaches was formed. Here, we describe and present findings from the participatory process to understand:

1) The contextual needs, what is currently working and not working for children of parents with a mental illness (drawing on data from the scoping stage),
2) The key elements and a conceptual understanding of best practice for COPMI (evidence review findings),
3) The desired practice elements of the approach to develop a model (codesign workshop findings), and
4) The conditions necessary to implement and trial the practice approaches (implementation design).

### Scoping Data Sources

A number of research activities were conducted in preparation of the co-design process aimed at understanding the local context and understanding international best practice. The following data were used and is now published: (a) a situational analysis of Tyrolean societal and service provision context in relation to families (46), (b) an analysis mental health care service uptake (47), (c) a mapping of mental health service usage in Tyrol (47), (d) a synthesis of the knowledge from the literature and international experts about what works, for whom, and in which context (48). These secondary data sources were narratively summarized to give an overview of the results of the scoping phase.

### Co-design Process Data

A series of six codesign workshops were conducted locally in 2018–2019, with live-video conferencing as needed, to develop the key design concept (49). The findings of the codesign workshops were documented in the workshop planning documents, transcripts from audio recordings of the workshops, as well as workshop materials such as slides and outcome documents. These documents described the aims and activities of the workshops, presented content delivered during the workshop, results and summaries of the decisions made, transcribed and translated audio recordings of the workshop discussions, and observations and reflections from the researchers participating in the workshops. Content analysis (50) was used to examine the key decision-making steps that led to the development of the practice model throughout the series of workshops.

While the workshops were mainly held in German, some aspects of the workshops were conducted in English to accommodate participation and delivery of content from non-native German speaking researchers (JP, MGo). All documented material, including audio transcripts, were translated to English, and examined by both a German speaker (IZK) and an English speaker (MGo) for accuracy and consensus in the content analysis (50).

## Results

The practice approaches were developed through a series of stages: (1) scoping; (2) co-design; (3) acceptance of the design; (4) aspects of feasibility and suitability of practice approaches to local context. Those stages are now described in detail below.

### Stage 1: Scoping—Identifying the Existing Context and Service System, Needs of Families, and International Best Practice

Firstly, scoping was conducted to understand the local context (46). The region of Tyrol is in the Western part of Austria, and geographically consists of many mountains and valleys. The population size is roughly 750,000 from which 140,000 (19%) are dependent children (0–18 years). Just over 85% of Tyroleans are Austrian citizens. Catholic religion plays the most important role regarding religious communities. With respect to economic structure, 50% of the population is actively working in paid employment, the remainder is either retired (20%), in education, or in other forms of activity (parental leave, household leading only, military service). Tourism industry accounts for 20% of the Tyrolean gross domestic product (46).

Data about the existing practices, barriers, and facilitators to support for children of parents with a mental illness in the existing service in Tyrol, Austria, were drawn from the scoping stage. Essentially this stage determined the scope of the unmet need and gaps in the existing service system.

#### Defining the Unmet Needs and Gaps

Service usage data indicated that the most parents in Tyrol were seeking treatment within the primary health system (e.g., medication prescription from a family doctor, GP), but the majority of severely ill parents were seeking treatment in the adult mental health inpatient hospital system (47). Support services, however, directly targeting children of parents with a mental illness were extremely limited in the region. Publicly funded mental health care or psychotherapy for children and adolescents were also limited, although privately funded outpatient psychotherapy/psychiatry was available for those families who could finance this themselves.

During the scoping stage, it was clear that there was an identified awareness and need to support children of parents with mental illness in the region. Stakeholder interviews identified many existing practice challenges in care for children of parents with a mental illness in Tyrol (46). These included:

- A lack of standardized identification and recording of parents with mental illness accessing treatment services. This included little or no documentation on the children of parents seeking treatment.
- A lack of standardized documentation, training, and education for professionals regarding identifying children who are living with a parent with a mental illness, particularly in talking with parents who are presenting to treatment services.
- A lack of awareness and practice guidelines in how to support children of parents with a mental illness and where to seek support for a family.

In terms of existing services provided, there was a recognition of the need to ask about a child's welfare if the parent presented to hospital or emergency services as unwell. However, there were little formalized processes of support services to refer children and their families for support, unless detrimental issues were identified. The main approach taken by adult mental health professionals involved contacting the child and youth welfare system or social worker within the hospital to address the crises needs of the family. This process of accessing support could lead to installing family support services, however, the system of support was triggered by referral due to an identified risk issue for the child (referral to child and youth welfare) (46).

Some social services were available including youth centers, parenting support programs, and mental health self-help groups for adults. Some voluntary support offers were also available such as “host grandmothers” and volunteers for tutoring in educational needs. One potentially relevant service was identified (“Kinderleicht”), specifically addressing the support needs of children of parents with addictive disorders. However, this service was small and only servicing one region of Tyrol. Issues were also identified across the region with equity of access to programs and support, with more service options available in urban areas compared to some of the rural regions of Tyrol (46, 47).

##### International Best Practice

Interviews with international experts in the implementation of family focused practice for these families found a number of key themes to understand more about the nature of the challenges and also enablers to practice in order to produce desired outcomes for children of parents with a mental illness. As described in (48), core components of programs included a focus on building strengths of parents in their parenting skills and helping children to understand parents' mental illness. The interviews also highlighted the interplay between practitioner, parent, and child outcomes; and the need for sufficient resources, such as training and supervision and organization support for family focused practice [see (48) for more detail].

##### Bringing in the Evidence Base

Brief scoping reviews were conducted in between the workshops to understand the core practice elements of the codesigned practice approaches, and to bring in international evidence for local adaptation. Key peer reviewed research articles were reviewed that covered practice guidelines and recommendations for practice and were expanded to using key literature searches in Medline, PsycINFO and Google Scholar for the terms “*identification”, “social support,” “collaborative practice,” “practice guidelines” “family intervention” “parents with a mental illness”* and “*children*” or “*children of parents with a mental illness”* or “*COPMI*.” In addition, the Village team of international researchers were each asked to review and explore known family focused interventions from their own and similar countries of origin to contribute to the existing approaches determined in the literature review. Core elements from the literature and selected best practice approaches were presented to workshop participants with a series of activities that enabled participants to select and discuss how to transfer the international evidence into the local context. See Appendix 1 for an example.

### Stage 2: A Series of Codesign Workshops With Local Community Stakeholders to Develop the Design of the Practice Approaches

Following a review of the key components of participatory codesign methodology (43, 45, 51–55), a series of workshops were designed by the Village Project team. The overall aim of the series of workshops was to develop practice approaches that were evidence-informed, suited to the context, are acceptable to local stakeholders, and feasible and ready for dissemination. As part of this process, it was anticipated that designated networks amongst stakeholders could be built to support the translation of the practices into local services, and a commitment and authorization by stakeholders managing local services could be gained to implement the codesigned practices in their own environment. The practice approaches and tools were developed to increase the identification of children and to support them in everyday life by strengthening networks of formal and informal support systems of the child and their family in Tyrol. A key focus of the design included a focus on including the “child's voice” in exploring and designing their “village” of support.

#### Participants

Key stakeholders were identified to participate in the workshops and included a representation from a variety of fields. The aim was to include a maximum of perspectives based on the findings from the scoping stages on identified potentially relevant organizations and professionals who may come into contact with these families (46, 47). Participants were then selected based on a number of criteria including field (practice, policy); sector; profession; target group; function (management etc) and gender. Another important consideration was also to ensure the number of participants did not exceed 18 per workshop to maintain a productive working atmosphere.

A total of 26 individuals representing 14 different local organizations participated across the six workshops. In addition to this, a total of 13 persons from the interdisciplinary research-partner team attended across the six workshops. On average, 16 community representatives and 4 research team members attended each workshop. There was higher representation from the health care sector, practice-focused professionals, and participants were more likely to work in the medical profession compared with other professionals (see Table 1 below). Adult mental health services were more strongly represented than others, more females than males and more participants were in middle management roles. Not all participants attended all workshops, but attending participants could nominate a proxy in their place if they wished.

**Table 1:** Characteristics of workshop participants.

**Sector (** ***n*** **)**		**Field (** ***n*** **)** ^**a**^	
Health care	11	Practice	14
Social care	6	Research	4
Education	2	Policy/Payer	4
Informal/voluntary	2		
Other	(1)		
**Profession (** ***n*** **)**		**Service sector (** ***n*** **)**	
*Medical doctor*	*4*	*Primary care*	*2*
- Psychiatrist	4	*Adult mental health*	*7*
- General practitioner	1	- inpatient	7
Nurse	1	- outpatient	5
Social worker	2	*Child mental health*	*3*
Psychologist	3	- inpatient	3
Pedagogue	1	- outpatient	3
Public health specialist	(1)	Children's service	3
Social scientist	(1)	Parental service	2
Peer worker	(1)	Service for families	3
Other	(1)	Not applicable	2
**Sex (** ***n*** **)** ^**b**^		**Function within organization (** ***n*** **)**	
Female	13–15	Top management	4–6
Male	6–7	Middle management	7–11
		Front line staff	3–6
		Not applicable	2

a *some stakeholders represented more than one field*;

b *number dependent on proxies that attended; brackets indicate that these categories were not represented in each workshop*.

#### The Workshops—Designing the Practice Approaches

The workshops included both presentations and group work facilitation exercises to develop up the concepts of the “It takes a Village” practice approach. Key decisions were made at each of the workshops to focus and consensus was sought on the design concept. Several facilitation techniques were used and these are described in (56).

The aims and key decisions of each workshop are described in Table 2 below. The workshops involved presenting international best practice examples and evidence on effective approaches. Workshop participants then identified options on how those might be implemented locally in Tyrol. The aim was to find a balance between evidence-based practice and feasibility within the local context and constraints (57, 58).

**Table 2:** Co-design workshop aims and key decisions in the development of the “It takes a Village” practice approach.

**Workshop** **(no. attendees)**	**Aims**	**Key decisions that resulted**
1 (*n =* 15)	Awareness of group participants and their relationship with the issue of COPMI. Development of a sense of identity as a codesign group. Understanding of the principles of open innovation and codesign and their role in this process. Introduction to the Village Project and a background introduction to the needs of COPMI from research and local scoping results. Presentation of three case vignettes outlining case journeys for COPMI within the region (information elaborated on from scoping) to identify areas of change.	Agreement on terms of reference. Agreement on rules for communication. Identification of key areas for change from the presentation of case vignettes of child focused care found in the scoping stage.
2 (*n* = 15)	Development of a shared vision. Familiarizing with a theory of change. Prioritizing areas for change.	Agreement made on the roadmap for the design of the practice approaches in the codesign workshop series. Agreement on common vision, assumptions and priority goals. Selection of max. nine prioritized areas for change.
3 (*n =* 17)	Identifying practice options for four prioritized areas for change around improving identification of COPMIs in adult mental health in Tyrol; based on proposed practice approaches in the literature and international expert interviews.	Agreement on options for transferring Phase 1—SENSE practices on identification to Tyrol (e.g., who should be asking about parent status, which questions to be asked, options on how parents admitted to hospitals can stay in contact with their child, options on how/where/when to talk with the child about the parental mental illness, options on how to address social resources around the child/family for the first time); agreeing on the stance (e.g., strength-based, acknowledging privacy, empathic and respectful, non-judgemental).
4 (*n =* 18)	Identifying practice options for the remaining five prioritized areas for change around improving support of children via a collaborative village approach (CVA); based on proposed practice approaches in the literature and in the international expert interviews.	Agreement on options for transferring Phase two—CVA practices on activating support around these children in Tyrol (e.g., how to refer the child/family to support program, which organizations could host the “facilitators” who would work with the child/family, which practice steps are involved in working with the children/families to activate support, which qualifications are required.
5 (*n =* 16)	Finalizing the practice concepts on identification and collaborative support from previous workshop. Identifying key aspects of the evaluation design (How to evaluate the change process as well as its results).	Agreement on the first point of identification, referral pathway and key practices of Village Facilitators in working with children/family as well as options for hosting the facilitator based on previous workshops. Agreement on inclusion/exclusion criteria, study design, options for outcome indicators. [see (48) for more detail of the outcomes]
6 (*n =* 13)	Defining feasibility, commitments and next implementation steps.	An agreed approach to practice, implementation and evaluation procedure is available including a commitment of organizations and persons to implement changes in their every-day practice.

### The Workshop Results

#### Key Decisions

Each codesign workshop was constructed to make key decisions about the development of the practice model, the evaluation, and the implementation to be delivered as part of the Village project, as outlined above.

In workshop 1, areas for change were selected from reviewing several case vignettes of existing practice drawn together from the scoping data [see more in (49)]. Areas for change from reviewing these vignettes were identified by the workshop participants. These were:

- Improving family communication about mental illness (parents and children).
- Improving education to families about mental illness.
- Asking parents about their children when seeking treatment.
- Providing psychoeducation to children in schools.
- Establishing adequate infrastructure for children to visit parents in adult psychiatry.
- Support contact between parents and children when parents are unwell and in treatment.
- Begin a conversation with families as early as possible when a parent is unwell:
  ∘ Inform children of their parent's mental ill-health.
  ∘ Develop standardized processes to identify social resources around the child.
  ∘ Develop guidance and knowledge of ‘good enough' parenting as an orientation for adult mental health professionals.
  ∘ Include development of a crisis plan in standard process of care.
  ∘ Include family members and children in the development of crisis plans and decisions.
- Primary health care to actively work with families of parents with a mental illness (provided the GP is aware of the parent's illness).
- Educate families/relatives about the importance of children needing support, as with physical illnesses in parents.
- Increase availability of social workers in adult mental health for family/child coordination.
- Adult Mental Health to refer families for support outside of psychiatry.
- Schools to provide supports for children of parents with a mental illness.
- Improve communication between organizations.
- Raise awareness in the community that children of parents may need support as much as children of parents with a physical illness.
- Organize mental health care earlier in a way that avoids the need for acute crisis care (avoid trauma for children).
- Improve information on available support in families, adult mental health, other relevant organizations and communities.

In Workshop 2, a consensus was reached about the common goal for the design:

*The Village approach promotes the healthy development and mental health of COPMI*.

Several preconditions and assumptions were agreed on for the design of the practice approaches. These included: the service provider has information of the parent's mental illness; there is increased help-seeking from families through better information and understanding of mental illness; and knowledge and awareness of mental health needs to increase in the community.

Workshop participants prioritized the areas identified in workshop 1 in terms of what is the best way forward to achieve the agreed vision. These were selected as follows:

1) All providers in adult psychiatry (for example psychosocial services) actively ask patients about their children/family situation.
2) Healthy caregivers and children are (kindly) informed about parental mental illness; talks take place as early as possible without hierarchy (child focused).
3) There are standardized procedures for identifying social resources around the child; caregivers are informed.
4) Contact between the affected parent and children is actively supported in the acute phase.
5) At each visit, a family contingency plan is prepared—mobilizing existing resources; caregivers. Children are involved in an age appropriate way. Decisions included; talks take place “at eye level.”
6) Families are actively invited by family doctors, supports if a parent is mentally ill.
7) Psychoeducation is developed and implemented for schools.
8) All providers know existing offers and their contents (for example, are better informed about child and youth welfare).
9) Support for children is actively organized and families are cared for continuously, while “normalization” is respected.

An agreement was made that the focus would be for activities within adult mental health—but other areas such as primary health and school system would be beneficial to include at a later stage. It was deemed that prioritized areas 1–3 were to be designed as part of Phase 1—sensitive identification (SENSE) and areas 4–9 were seen as steps within Phase 2—the collaborative village approach (CVA).

#### The Design Concept (Results From Workshops 3–4)

**Figure 1:**
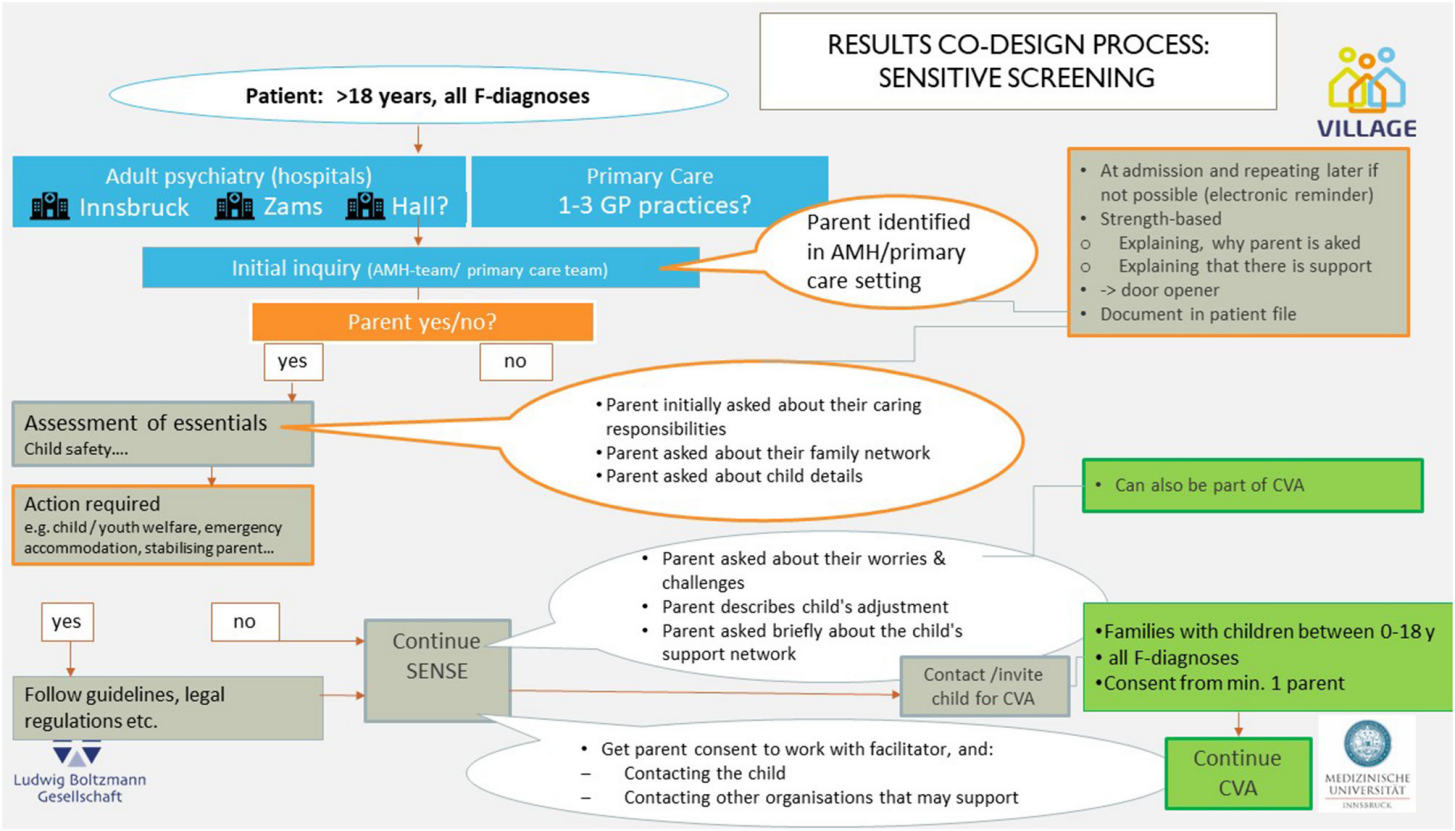
SENSE (Identification) pathway. CVA, collaborative village approach; SENSE, sensitive screening; GP, general practitioner.

**Figure 2:**
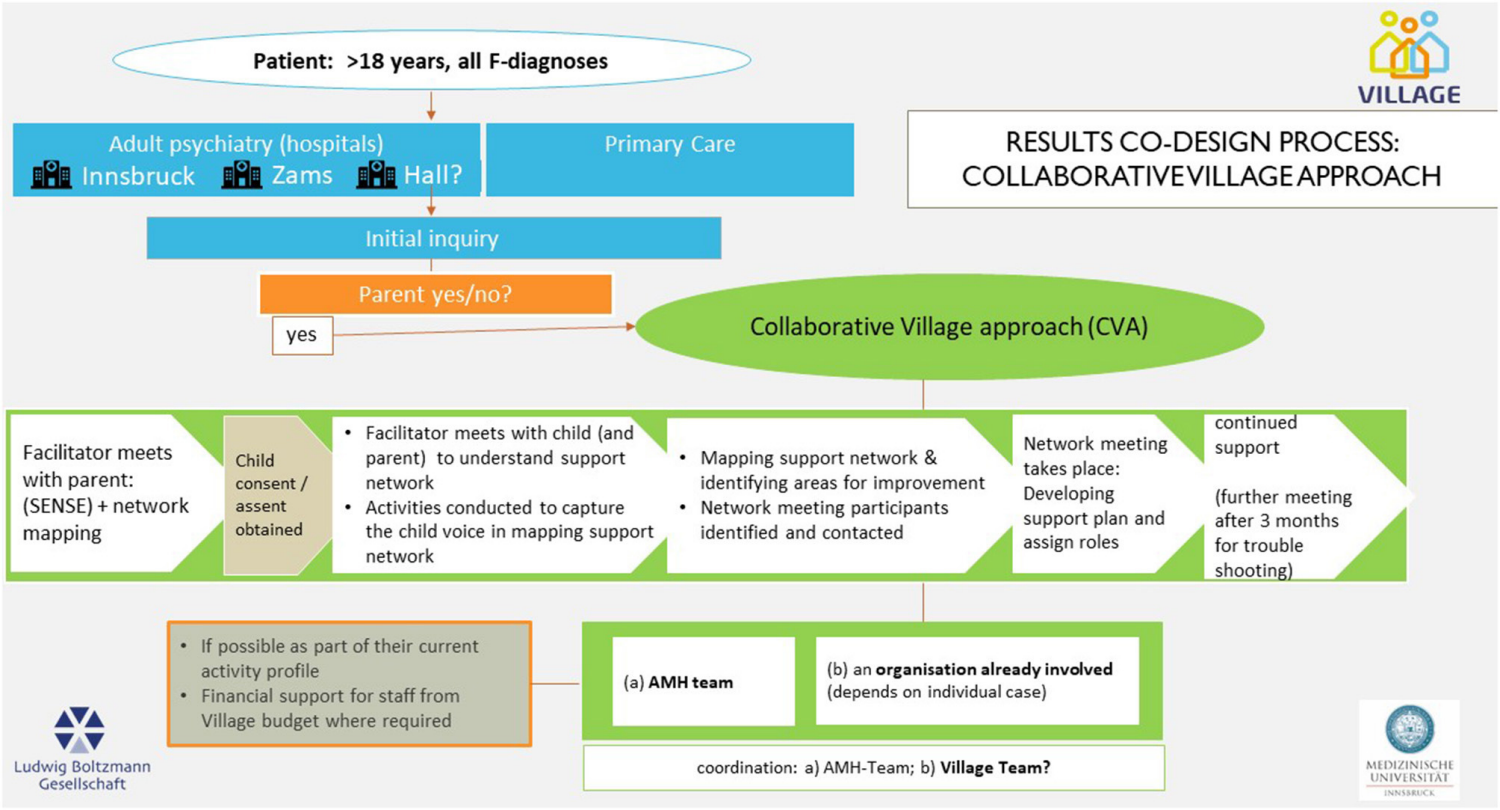
Pathway for the Collaborative Village Approach (CVA).

The product at the end of the workshop series included two practice models: (1) a visualization of a pathway for the identification of children of parents with a mental illness—a standardized and systematic SENSE process in selected hospital adult mental health and primary care institutions (Figure 1); and (2) a visualization of the process of establishing both informal and formal support (the Village) for children of parents with mental illness and their families—the Collaborative Village Approach (CVA) (Figure 2). These draft concepts were agreed to by the workshop participants as the primary design outcome, that would be implemented and evaluated in the next stage of the research project. Some details (e.g., with regard to coordination responsibilities) remained unsolved at that point in the design process (indicated by question marks in the figure). For some points within the pathway, options were specified.

**Figure 3:**
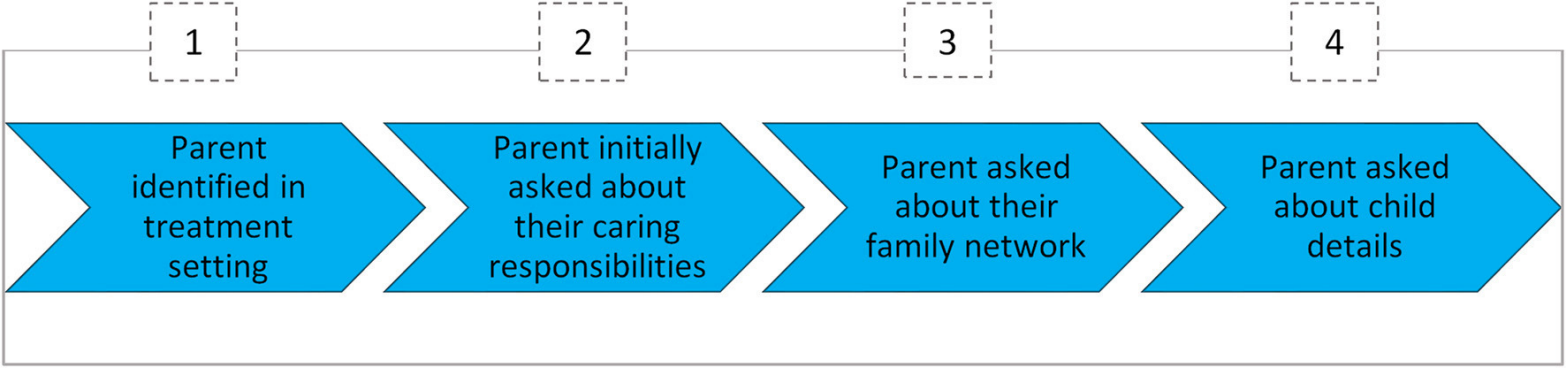
Practical elements of SENSE phase 1.

**Figure 4:**
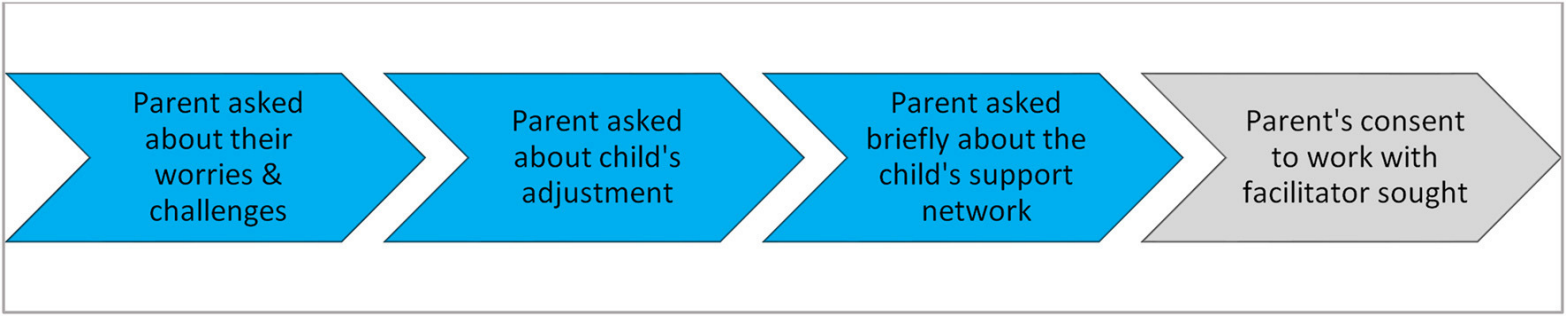
Practical elements of SENSE phase 2.

**Figure 5:**
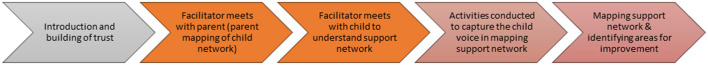
Practical elements of CVA Phase 1.

**Figure 6:**
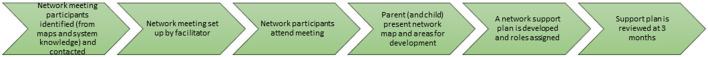
Practical elements of CVA Phase 2.

The process steps were unpacked separately as part of the workshop process, and are shown in Figures 3–6.

**Key Steps** in the practice model that were agreed to were as follows:

1. Identifying parenting status and child and family characteristics and responsibilities (SENSE 1; Figure 3).
2. Exploring with a parent about the child's adjustment—strengths and challenges (SENSE 2; Figure 4).
3. Developing a shared understanding with parents and children on the day to day life of the child and the supports in place and needs to strengthen these supports (CVA 1; Figure 5).
4. Develop a support plan to strengthen and maintain the child's supports through a network meeting (CVA 2; Figure 6).
5. Review the support plan, troubleshooting and addressing issues for sustainability into the future (CVA 2; Figure 6).

### Sensitive Screening/Identification of Children Living With a Parent Who Has a Mental Illness

#### SENSE 1: Identifying Information and Building Trust

The goal of phase 1 of the SENSE approach is to identify whether a patient with a mental illness has children and is therefore a father or mother (Figure 3). Identification questions are intended to be used during admission or during a visit of the treating physician or during a visit to the general practice.

One initial outcome required from this SENSE approach is the recording of the **parenting status** of the adult patient, their **family caring roles**, and their **children's gender** and **age and living situation**.

#### SENSE 2: Short, Goal-Focused Conversation About Parenting and the Daily Life of the Child

The outcome of phase 2 of the SENSE approach (Figure 4: Practical elements of SENSE phase 2) is a more in-depth conversation with parents about their parenting **strengths and challenges**, strengths and vulnerabilities for their **child's adjustment**, and a brief understanding of the existing **child's social support network**. The parent could also be asked about any immediate needs and wishes they may have for enhancing the strengths of their child, or in relation to their parenting strengths and challenges. Sensitive, open questions to understand the current living situation of the parents and the child are important here, for example, *Can you tell us a little about your parenting and caring roles at the moment in your life?*

### Enhancing the Social Network: The Collaborative Village Approach (CVA)

The idea of CVA is to help build a day-to-day life that ensures the best possible support for the child/youth in their local support network, or “village” and thereby promote the healthy development of children of parents with a mental illness. This should be driven by parent and child in partnership and supported through conversations with the Village Facilitator. In this part the village facilitator takes on a key capacity building and curious role. The Village Facilitator works together with the family and their social network to strengthen the social support for the child. Working directly with the child and seeking their perspective is a key component of the CVA.

#### CVA 1—A Shared Understanding the Support Network of the Child

The first step of the CVA approach involves the Village Facilitator engaging with a parent for the first time in the role of the Village Facilitator (Figure 5). The primary focus of this first interaction is to build engagement and the beginnings of a collaborative relationship to promote the well-being of the parent's child. As part of this interaction, a series of activities and questions are asked to learn about the child's social network from the parents' perspective. The aim is to develop a common understanding of the child's everyday life, existing support and possible gaps and potential for improvement between the parent and Village Facilitator. The role of the Village Facilitator is to identify the parent's view of the child support network and ask specific questions when needed to understand missing persons or institutions who are capable to close potential network gaps for the child.

The next step in this phase is for the facilitator to support the children to contribute their understanding of their social network. This step helps create a common understanding with parents and children about the child's everyday life and existing support, as well as to identify what is needed to improve the situation. The activities described are aimed for children from 4 years of age. The aim of the activities is to hear directly from the children about their support. This activity visualizes the existing networks and identifies gaps in support. Ultimately, the child should help define what their “village” looks like and this network should be made visible.

Following an analysis of the parent and child support network results, a family meeting is conducted to help develop a shared understanding of the support network and identify areas for enhancement or improvement. The idea of this step is to develop a common idea of how the subsequent network meeting in CVA 2 should be organized.

#### CVA 2—The Network Meeting and Support Plan Is Developed and Reviewed

The concept of the network meeting (Figure 6) is derived from the “Family Group Conferencing” practice (other common names: Social Network Conference, Family Group Conference, Family Council, Relatives Council). The concept for our CVA network meeting was informed by early developments in family conferencing in New Zealand and has since then been applied in a wide range of fields (e.g., child protection, domestic violence, youth justice) including mental health (59–61).

Through an independent coordinator (in our case “Village Facilitator”) informal and formal support systems are brought together, while at the same time the family and especially the children are encouraged to take responsibility for decision-making. In other words, the Village Facilitator is responsible for the process, but not for the outcome of those meetings. The underlying ethos of the network meeting is based on the principle that the family and its social network are capable of finding their own solutions to support children with mentally ill parents (they have control over the solutions and are recognized as experts in their own lives).

The role of professional service providers and community members is to facilitate and resource plans and decisions that are consistent with securing and supporting a child's resilience in their daily life. The focus is to support the child's day to day activities, and the provision of practical, emotional, and social support. At the end of the network meeting, there should be an agreed support plan which enhances the daily life of the child, both from formal and informal support providers for the child. Following 3 months of implementation of the support plan, the plan is reviewed for any future refinements. At the end of a 6 months phase of engagement, the idea is that the work of the village facilitator is handed fully over to families and support personnel to lead and maintain the network of support where needed.

### Theoretical Basis of the Approach

As discussed in the workshops the theoretical approach and stance is a core part of family interventions for families where a parent has a mental illness. It was agreed that the practice approaches are delivered with the following theoretical perspectives in mind:

1. Motivational interviewing
2. Capacity building approaches for families and practitioners
3. Consideration of the social determinants of health
4. Working within an understanding of the sociology of childhood
5. Focusing on building self-regulation skills and promoting self-determination and choice in families

### Core Practice Principles

The Practice Approach is built on the following principles of practice:

- Orientation on strengths of the family (members) instead of weaknesses
- Recognition of the decision-making competence of parents and children (building self-determination)
- Trauma Sensitivity: Awareness of the effects of traumatic events in families and children and creating an atmosphere in which all persons feel safe, welcome, and supported

The aim of the described practices is to develop a sense of trust and a feeling of confidence for the concerned parents and children. All elements of the described practice approach open up the possibility that the parenting experience with a mental illness and growing up in a family where one parent is mentally ill will be normalized (with the experience of not being alone) and recognized. The focus is also on a non-judgmental, interested stance toward families, which helps to create a trustful and supportive atmosphere for parents and children and which helps mental health professionals, general practitioners, or village facilitators to have a meaningful conversation with parents and children.

Another central principle of all the practice steps described above is that, whenever possible, the viewpoint of the children and the parents is integrated into all processes and decisions. The perspective of the families concerned serves as an essential basis for understanding their needs and developing a common social support for the children.

### Stage 3: Acceptance of the Design

Commitments for participating in the different practice steps were sought and documented in Workshop 6. A commitment for the SENSE approach was obtained and these sites would serve as the pilot sites—one hospital inpatient based (Innsbruck), 1 day clinic based (Zams). The possibility of identifying parents in primary health through GP practices was also suggested. The CVA approach was seen to be a process outside of psychiatry, in community services; with the exception of the day clinic (Zams) that proposed a model where CVA process could be delivered as part of the routine treatment team.

### Stage 4: Aspects of Feasibility and Suitability of Practice Approaches to Local Context

At the completion of workshop 6, participants undertook an activity to identify barriers and enablers for the implementation of the codesigned practice approaches. In terms of where the practice approaches could take place, participants deemed SENSE could feasibly be delivered in adult mental health services or general practice clinics, without the need for additional resources or costs. Participants stressed that it would require, however, equipping existing staff with the procedures and supportive structures to undertake SENSE and refer parents with mental illness to the Village Project. Structured documentation and leadership were deemed to be important to support staff to undertake SENSE. Primary health care needed structured questions, and Adult Mental Health needed question prompts documentation to ask questions that identify children of adult patients as part of routine practice.

For the CVA process, workshop participants indicated that in most situations this process was outside adult psychiatry and would need to be resourced through additional funding. Although one adult mental health service identified they could embed a village facilitator within the treating team, if they were commissioned and funded to do so (the hospital in the village of Zams, Tyrol). Clear referral pathways to CVA were needed, as well as knowledge of the possible support network options available in the region needs to be clearly documented.

Several uncertainties to the implementation were identified by the workshop participants. Concerns were raised regarding a lack of time, money and staffing resources to deliver the practices; lack of willingness from informal care providers; lack of suitable physical resources and infrastructure available; lack of organizational support for village facilitation role; difficulty co-ordinating attendees for network meetings; difficulties seeking informed consent from families; language and communication barriers; and skills in talking sensitively with parents and children. Several options were discussed as part of the activity that might help overcome these situations (see Appendix 2 for more information).

- Finally, workshop participants indicated the willingness of their organizations to implement the practice approaches.
- Commitments were made to implement the SENSE in two Adult Mental Health settings and potentially 1–3 primary health care settings.
- Commitment to take part in CVA network meetings in 11 out of 14 participating organizations.
- Expression of interests to provide staff for village facilitator role in 4 represented organizations.
- Commitment to participate in the implementation check-ins (local implementation team, multi-agency implementation team, advisory board) by organizations that will implement practice changes.

## Discussion

This paper showcases a process of intervention design to address a gap in service delivery for children of parents with a mental illness in Tyrol, Austria. The intervention “It takes a Village” approach consists of evidence-informed and codesigned practice elements, developed with people with lived experience in practice and also with those living with the challenges of mental illness in the region (3). The approach includes elements of practice that assist adult treatment providers to sensitively identify parents of dependent children who may be seeking treatment for their mental health challenges (SENSE). The second component consists of practices and steps for facilitators to enhance the “village” of support for a child living with a parent with mental health issues, and includes a focus on informal and formal support structures as well as understanding the parent and child's perspective on the child's daily life (Collaborative Village Approach, CVA).

The “It takes a Village” practice model, as outlined in this paper, is built around a participatory process from all areas of the project, including in understanding (1) the contextual needs, what is currently working and not working for children of parents with a mental illness (scoping), (2) key elements and a conceptual understanding of best practice for families where a parent has a mental illness (evidence review), (3) practice elements of the approach to develop a model (codesign), and (4) understanding the conditions necessary to implement and trial the practice approaches (implementation design). Alongside this, was the development of an evaluation logic and realist approach framework to design the outcome measures of the evaluation of the village approach (48). From this process, we achieved a high agreement from stakeholders to trial the practice approaches, where relevant, in their organizational setting.

The process draws on approaches outlined in the implementation science field. Here we have applied best practice from implementation science in applying principles of codesign and a series of structures and strategies to help integrate best practice evidence into “real world” settings (62). We have utilized a participatory design approach where those involved in delivering the intervention or using services shape the evidence of what works into a practice approach suitable for their contextual setting. These approaches are becoming fundamental to the transfer of innovation that when applied involve changes to practice, particularly in mental health settings (42). A paradigm shift toward recovery-oriented practice, from a predominantly bio-medical focused one has encompassed a strong focus on consumer involvement in service design and resulted in a range of successes in service delivery approaches. This shift in service delivery has been found to occur more successfully when there is a whole of organization approach involving organizational leadership as well as consumers with lived experience in the design and support for the delivery of these new methods of practice (63). We expect the process described for development of the practice approaches in Tyrol will show similar ease in the transfer to practice and service delivery for families locally.

As shown in the workshop series, the codesigned practice approaches were developed on the evidence base for interventions and supports for families where a parent has a mental illness. Steps outlined for the practice approach in this paper have similarities with practice elements outlined in other well-known evidence-based interventions such as Let's Talk About Children (38, 64), Family Talk (65), Parenting Well (66, 67), and Social Network Conferences (38, 68), and other evidence-based practice elements described in the research (4, 11, 69).

A fundamental basis to the “It takes a Village” practice model is drawn from the use of motivational interviewing techniques to assist in outlining rapid engagement techniques that can support practitioners in talking with parents and their children. Motivational interviewing skills uses various communication techniques to improve a person's self-efficacy or sense of their own capability, and enhances their motivation for changes through a focus on a person's desired behaviors (70–72). Because of this, motivational interviewing has parallels with the promotion of self-determination and self-regulation in a person (73), two areas of change that has more recently been linked as core elements for families benefitting from family interventions (5). Motivational interviewing skills also prove useful in managing parent ambivalence or engagement issues in child and family social work (74). Similarly, self-regulation skills are also proving useful in working with parents with mental illness for engagement in parenting support programs (75). Interestingly, they are also now being considered as a strategy in supporting practitioners in the change process to implement new practice approaches themselves (76).

A common criticism of selection prevention approaches for children of parents with a mental illness has been a lack of theory or conceptual framework in the evidence base of family interventions for these families (77, 78). This presents a problem for not only the design of evaluation or outcome studies, but also in understanding the assumptions underlying the mechanisms of change associated with mental health, family functioning, and child development that selective prevention programs are usually targeted toward (79). Some family evidence-based interventions in this area though report strong theoretical foundations associated with strengths-based, recovery-focused or resiliency frameworks (65, 80, 81). Drawing on the international evidence, the codesign workshop series described in this paper was able to explore the theoretical perspectives and evidence base to formulate practice approaches built on concepts of being strengths-focused and trauma-informed; built on theories of self-determination and self-regulation; and integrating an understanding of social determinants of health, and the sociology of childhood in its design. These perspectives are operationalized in the designed practice approaches through the stance and curiosity lens of the approach. This encompasses a focus on the “how” a practice is delivered as well as the “what” in terms of components of the practice approach.

This essential aspect of the designed practice approaches is reinforced through the questioning and engagement stance adopted by those working with these families in the delivery of the practice approaches. The stance highlights the values that underlie the practical action and determines *why* a professional may do something in a certain way when working with parents and their children. The principles of practice outlined in the stance include: (1) An orientation on strengths of the family (members) instead of weaknesses; (2) Recognition of the decision-making competence of parents; (3) Integration of the child's voice and perspective as a fundamental basis to the support plan design; and finally, (4) cultural and trauma sensitivity in practice. The focus is also on a non-judgmental, interested stance toward families, which helps to create a trustful and supportive atmosphere for parents and children and which helps a clinician, general practitioner, or village facilitator to have meaningful conversations with parents and their children. This focus is not new though to family interventions for children of parents with a mental illness. These are reported components of interventions such as Family Talk (65), Let's Talk about Children (5, 38, 64) and Family Options (80, 81).

A core part of the participatory process of the design of the Village approach was in designing a practice model that is acceptable and feasible for implementation in the local context. Participants were able to prioritize areas for change based on a thorough scoping stage, and also adapt the evidence base to the local setting of what might work within the region of Tyrol, Austria. The stakeholders with decision making abilities or policy influences were also able to indicate an agreement and willingness to implement the practice approaches at the completion of this codesign process, securing the beginnings of the next stage of the research project for “real world” implementation and evaluation. This participatory process has many advantages but is particularly encouraged in the development of innovations to help address the lag in efficiencies to translation to practice of evidence of what works, particularly for the reduction in burden of disease in public health approaches (42, 82).

Community stakeholders, in this study, however, identified that even with a process of codesign, there still remained challenges and uncertainties to the implementation of the practice approaches in the local setting. These barriers were believed to require organizational support to be overcome in the relevant practice settings. They included an allocation of time, resources, and funding to support the practice approaches to be delivered; alongside various skill-based training supports, policies and procedures to undertake the identification process (SENSE); and a flexible approach to the delivery of network meetings and requirements of informal and formal support providers. While not new, the application of family focused practice in mental health care settings continues to be accompanied by significant challenges in its implementation (21, 83). The integration of implementation science principles that aid in creating drivers to support practice change is becoming an important vehicle for effective translation to practice of evidence-based interventions in this area (44, 84, 85), as well as working in partnership between researchers, policy makers and service providers as part of support and sustaining change (86).

Selective prevention strategies, such as parenting or child focused interventions for families with mental health challenges, remain an effective public mental health strategy to improve child outcomes for children of parents with a mental illness (12, 87, 88). Such interventions have been shown to reduce the relative risk of a child developing the mental illness as its parent by about 40% (22). It is expected the “It takes a Village” model, which draws heavily on other effective interventions, will also improve outcomes for children (48). Interventions such as this—that focus on a two-generational approach (a parent and the child)—and on drawing together or improving elements within a child's daily life or ecology of influence—have also been shown to be effective in other selective prevention programs such as in child welfare with multi-stressed families (44, 89–91). Core to positive outcomes in research in this area, however, rely on program fidelity and implementation support strategies to that ensure program elements are delivered as intended (92, 93).

Equity of access to mental health-care, particularly for selective prevention approaches, remains a significant global challenge (21). Of note, in the design of the “It takes a Village” approach, implementation of the practice approaches were designed for primary health as well as adult psychiatry, a decision we expect will improve access for a number of parents who might be seeking medication support from their general practitioner only. We know from the scoping analysis that this will contain a significant number of Austrian families (47). While providing options for improving reach of the research study, this implementation approach is also in line with a focus on more community based and stepped care model of mental health care, whereby people have access to treatment outside hospital based mental health services (12).

In terms of limitations, it must be noted that this is not a study of the effectiveness of the practice approaches. While it broadly is expected to produce the desired outcomes for children of parents with a mental illness, there is evidence to suggest that family interventions in Austria for vulnerable children can have poor uptake (94). Therefore, there are significant unknowns about how these practice approaches may work in socio-cultural norms of Tyrol, and an understanding of the impact on child outcomes is yet to be determined in the project. We anticipate though, that with thorough consultation and design with local stakeholders as well as an understanding of best practice and implementation from the international literature, we are positioning the “It takes a Village” approach with solid foundations for achieving positive outcomes for families where a parent has a mental illness in Austria. A realist framework is being utilized in the evaluation of the practice approaches in Tyrol (3, 48) and it is anticipated that this selective prevention approach will be effective in improving the social and emotional well-being of children and their parents with mental health challenges.

In conclusion, the paper outlines a key process to developing evidence informed changes to practice and service delivery in mental health care for families. The participatory process itself, with key stakeholders, is a vital element in developing the translation to practice to suit local contextual needs. This is necessary to ensure effective elements of service redesign can meet and address existing gaps in care to intervene in addressing the intergenerational transmission of mental illness within families. Future studies in this project, however, will ultimately determine the direct benefits for families, practitioners, and the service system in Austria.

## Data Availability Statement

The original contributions presented in the study are included in the article/Supplementary Material, further inquiries can be directed to the corresponding author/s.

## Ethics Statement

The studies involving human participants were reviewed and approved by Monash University Human Research Ethics Committee. The patients/participants provided their written informed consent to participate in this study.

## Author Contributions

MGo led the analysis with IZ-K but all authors contributed to the analysis. MGo led the manuscript preparation and all authors were involved in editing the manuscript. All authors contributed to the design of the study and the collection of data.

## Funding

The Village is a research project funded by the Austrian Federal Ministry of Health—Science and Research through the Open Innovation in Science Center at the Ludwig Boltzmann Gesellschaft GmbH, hosted at the Medical University of Innsbruck, with collaboration of Co-Investigator institutions.

## Conflict of Interest

The authors declare that the research was conducted in the absence of any commercial or financial relationships that could be construed as a potential conflict of interest.

## Publisher's Note

All claims expressed in this article are solely those of the authors and do not necessarily represent those of their affiliated organizations, or those of the publisher, the editors and the reviewers. Any product that may be evaluated in this article, or claim that may be made by its manufacturer, is not guaranteed or endorsed by the publisher.
