# Supplementary material for: Development of an Evidence-Informed and Codesigned Model of Support for Children of Parents With a Mental Illness— “It Takes a Village” Approach
Source: Front Psychiatry. 2022 Jan 31;12:806884. doi: 10.3389/fpsyt.2021.806884 (PMC8841827; doi:10.3389/fpsyt.2021.806884)
Supplement: Supplementary file 1 [file Data_Sheet_1.docx]

**Appendix 1: Questions to workshop participants on 4 prioritised topic areas**

Topic 1: All providers in adult psychiatry (hospital, psycho-social services etc.) actively ask patients about their children / family situation

1. Key Questions Analysed:
   - 1. Who normally asks?
     2. Where and when are parents asked?
     3. What are they generally asked about – what information?
     4. How is information recorded?
     5. Preconditions to helping the parent participate
     6. Preconditions to helping the practitioner deliver the practice

Topic 2: There are standardized procedures for identifying social resources around the child; Caregivers are informed

1. Key Questions Analysed:
   - 1. What is generally asked about in the identification of social support for the child/family?
     2. By whom, with whom and How is the initial assessment usually made (or recommended)?
     3. How might information be recorded?
     4. What minimum detail is needed to assist in a referral?
     5. What is the minimum information provided to caregivers (or what considerations need to be thought about)?
     6. Are there any important considerations to support the initial assessment (e.g. info about available referrals)?

Topic 3: Healthy caregivers and children are (kindly) informed about parental mental illness; Talks take place as early as possible (at eye level)

1. Key Questions Analysed:
   - 1. What do children want to know about mental illness?
     2. What do adult family members and caregivers want to know about mental illness?
     3. Who normally informs?
     4. How is knowledge normally transferred?
     5. What resources (books, videos, pamphlets…) are available?

Topic 4: Contact between the affected parent and children is actively supported in the acute phase

1. Key Questions analysed:
   - 1. How is contact generally maintained when a parent is unwell and hospitalized?
     2. What information is provided to the children?
     3. Preconditions to helping the parent and child participate?
     4. Preconditions to helping the practitioner and organisation actively support the practice?

Topic 1 Summary of the evidence – Asking patients about their children in adult mental health

| What to ask? | Do you have a child? Yes/NO  Where is the child?  What is your child’s age?  How many children do you have?  Gender of the child  Who can care for the child + record two contacts  Actively show interest and to offer help – and further discussions later (open the door to more conversations)  Offer support to parent – can I call someone for you (your child’s caregiver or your child) |
| --- | --- |
| How to ask? | Explain to the parents WHY you are asking  Reassure parents that fears are normal.  Give them the reason as to why you are asking and show interest.  Reassure parent that there is support.  Also mention what children want and like.  Signpost for parents the possible offers available (‘plant the seed’)  Normalising the struggles and promote strengths  It is common situation that children can worry about their parents. |
| When to ask? | Ask at the beginning but may get a no response or lack of response at first  May not be able to ask at first because of acuity or intoxication, but practitioners need to be reminded to continue to ask. |
| Documentation needed | Document in the patient record  Reminder to document, if first missed.  One click within existing documentation system |
| Remaining open questions | WHO has the right to access all of the info in the patient record  Who should ask:  The role of the doctor at admission, and then later be the care staff?  Where there is continuity of care or being present around the patient? (is this the case manager/ or station manager)  Preferred same person?? |

**Appendix 2: Barriers and ways to overcome these barriers identified by workshop participants.**

| **Potential barriers** | **Ways to overcome** |
| --- | --- |
| **Provision of informal care**  Concerns were raised about finding suitable people who are willing to provide informal support to families - because it may be too much responsibility and / or time required; informal carers may also be worried about the long-term impact of their caring role on the relationship with the parent (e.g. if it’s a close relative of the parent) | -Work with voluntary workers (Ehrenamtliche) which will then have a clear delineated role (e.g. godparents role)  -Barrier may in fact not be as big if the concept of informal support is seen more flexibly (informal support may be a very small task – e.g. the neighbour checking in once a week) but not replacing the parent or taking over the parenting role entirely |
| **Space/physical infrastructure**  If network meeting and meetings with child / families to prepare do not take place in the hospital; it may be difficult to find adequate rooms. | -Several of the organisations who expressed interest in being a potential facilitator could use rooms within their organisations (e.g. school social worker in school, Elternbildung, Sozialberatung der Caritas, Eltern-Kind Zentren, Erziehungsberatung); there are some organisations that have offices spread across Tyrol (e.g. Caritas) where arrangements may be found to use their rooms. |
| **Language issues**  May be a barrier if activities take place outside hospital because then the hospital online interpreter service cannot be accessed | -There are some resources available in the school and social care sector that provide translation services, however they have low capacities and some currently face budget cuts (e.g. school area: mobile Integrationsteams, Frauen aus allen Ländern,…)  -Village facilitators may themselves speak several languages (e.g. Greek) and it could be tried to train people as facilitators who have specific language skills (e.g. migration background) |
| **Working sensitively with children**  Difficulties in working with children and understanding mental illness and the issues for families | -Training, psychoeducation could be provided to facilitators |
| **Role of facilitation tasks**  Provision of facilitation task may be outside the activity profile of an organisation who expressed interest but would then not be allowed to do facilitation in all cases (e.g. services that are part of child and youth welfare can only take cases where the child and youth welfare is already involved; school social workers can only facilitate cases which involve a child that is visiting a school they serve; Frühe Hilfen can only work with families with children up to age 3; Elternbildung when there is a mental illness in the family) | -Having facilitators trained from a variety of organisations so that the chances are high to find a facilitator out of an available pool that can take over the case  -Extending the activity profile of some services (e.g. Pro Mente – Rehab – change Leistungskatalog) |
| **Lack of resources**  Some people / organisations who expressed interest won’t be able to integrate facilitation into their existing routine work (e.g. some would need to increase working hours which would need to be paid extra)  Adult Mental Health (AMH) was worried that they have limited time and resources within AMH team and limits to what psychiatry could do (SENSE was deemed to be possible within the existing role of AMH in Innsbruck and Zams, but the CVA process needed to be funded) | - Fund from village budget  - Try to train as many facilitators as possible from organisations that can provide the required activities as part of their routine work (e.g. school social workers would be able to run network meetings as part of their usual work)  - Structured documentation and leadership were deemed to be important to support staff to undertake SENSE |
| **Network meeting coordination**  Difficulties in coordinating network meeting (needs to be done relatively quickly, may be difficult to find a time when all network partners can come together) | - Handling these meetings flexibly (e.g. separate meetings into two); very likely there will be many cases with low number of network partners required |
| **Informed consent**  AMH were concerned about consent procedures and who would undertake these. While they saw the importance of assessing the patient’s ability to consent, they also were concerned to separate the treating team from the process of seeking informed consent. |  |
| **Child safety issues**  Key considerations included understanding how to manage the child safety issues that might present |  |
| **Sensitive questioning skills**  Staff may not be familiar with how to do; Lack of staff with facilitator skills | Primary health needed structured questions, and AMH needed question prompts.  Skills of the village facilitator were discussed: to be able to discuss sensitive issues with a parent and child, to work with professionals and the community; and to be able to be alert to and manage child safety issues and issues between a parent and child. It was also important to consider what the village team would do if a parent did not allow a child to participate, and how to manage that. |
| **Logistics**  Barriers in coordinaton and communication processes | Clear referral pathways to CVA was needed, as well as for an understanding of the possible support network available in the region needs to be clearly documented. |
